# Tissue-resident macrophages and renal diseases: landscapes and treatment directions

**DOI:** 10.3389/fimmu.2025.1548053

**Published:** 2025-03-31

**Authors:** Zhuojian Qu, Jinjin Chu, Shuyu Jin, Chunjuan Yang, Jie Zang, Jin Zhang, Donghua Xu, Min Cheng

**Affiliations:** ^1^ School of Basic Medicine, Shandong Second Medical University, Weifang, China; ^2^ Center of Medical Research, Weifang People’s Hospital, Shandong Second Medical University, Weifang, China; ^3^ School of Pharmacy, Shandong Second Medical University, Weifang, China; ^4^ Department of Rheumatology, Weifang People’s Hospital, Shandong Second Medical University, Weifang, China

**Keywords:** tissue-resident macrophage, renal injury, inflammation, fibrosis, kidney immunity

## Abstract

Tissue-resident macrophage (TRM) is a specialized subset of macrophage that resides within specific tissues and organs. TRMs play crucial roles in resisting pathogen invasion, maintaining the homeostasis of the immune microenvironment, and promoting tissue repair and regeneration. The development and function of TRMs exhibit significant heterogeneity across different tissues. Kidney TRMs (KTRMs) originate from both embryonic yolk sac erythro-myeloid progenitors and the fetal liver, demonstrating the capacity for self-renewal independent of bone marrow hematopoiesis. KTRMs are not only essential for the maintenance of renal homeostasis and the monitoring of microvascular environment, but contribute to renal injury due to inflammation, fibrosis and immune dysfunction in kidneys. In this review, we summarize currently available studies on the regulatory role of KTRMs in processes of renal injury and repair. The altering effects and underlying mechanisms of KTRMs in regulating local tissue cells and immune cells in different renal diseases are reviewed, primarily including lupus nephritis, diabetic nephropathy, renal fibrosis, and renal carcinoma. Understanding the plasticity and immune regulatory functions of KTRMs may offer new insights into the pathogenesis and the exploration of therapeutic strategies of kidney diseases.

## 1 Introduction

Macrophage is a crucial immune cell subset in mediating innate and adaptive immunity. There are two types of macrophages, namely tissue-resident macrophage (TRM) and wandering macrophage. They are involved in various pathophysiological processes, such as immune reaction, immune tolerance, inflammatory response, fibrosis, angiogenesis, and tumorigenesis (1). TRMs that reside in different tissues and organs are characteristic of heterogeneity, plasticity, and specificity, originating from erythro-myeloid progenitors (EMPs) in the embryonic yolk sac (YS) independent of transcription factor Myb (2, 3). TRMs are integral to host tissues and function with specific tissue cells during the processes of immune surveillance, inflammation regulation, tissue injury and repair, and maintenance of homeostasis (4–6). Two distinct developmental programs govern the differentiation of EMPs into TRMs. In the YS, YS-TRMs serve as precursors of microglia without the formation of monocyte intermediates. Conversely, fetal monocytes (MOs) are produced when EMPs migrate and colonize the fetal liver (FL), which serve as precursors of TRMs in other specific tissues, including kidney TRMs (KTRMs) (7). An increasing number of studies have demonstrated that TRMs play crucial roles in the regulation of organ and regional immunity as well as microenvironment homeostasis. For instance, enhanced apoptosis of visceral adipose tissue (VAT)-resident macrophages induces systemic insulin resistance in non-diabetic mice via Toll-like receptor (TLR) 4 and danger signaling-associated protein high mobility group box 1 after myocardial infarction injury (8). Kupffer cells protect liver from ischemia-reperfusion injury via heme oxygenase-1 (HO-1) (9), while lung-resident macrophages exacerbate remote sterile lung damage by promoting inflammatory response through the receptor for advanced glycation end products (RAGE) and the epidermal growth factor receptor (EGFR) signaling pathway (10). All these findings have suggested TRMs are not only the integral components of the immune system, but exert diverse effects on the regulation of organ and regional immunity with significant tissue specificity.

Kidney disease has emerged as a major problem globally (11). Macrophages are crucial for immune surveillance and the maintenance of renal homeostasis (12). Kidney macrophages exhibit heterogeneity consisting of bone marrow-derived macrophages (BMRMs), also referred to as infiltrating macrophages, and embryonic-derived self-sustaining KTRMs (13). Previous studies have primarily focused on the role of BMRMs in renal diseases. Increasing evidence has suggested KTRMs also play a significant role in the regulation of homeostasis in kidney independently or synergistically with BMRMs. In this review, we aim to elucidate the characteristics and functions of KTRMs, as well as the effects and mechanisms in the regulation of renal injury and repair in various renal diseases, to provide an updated insight into the pathogenesis and therapeutic strategies for renal diseases.

## 2 KTRMS

### 2.1 Origin, localization and maintenance of KTRMs

KTRMs originating from MOs are characterized by CD45^+^, CD11b^low^, F4/80^high^, and Ly6C^-^ (7). Some transcriptional regulators are activated in KTRMs exhibiting tissue-specificity, including the Aryl hydrocarbon receptor (Ahr), nuclear factors of activated T cells 1 and 2 (Nfatc1 and Nfatc2), and interferon regulatory factor 9 (Irf9) (14). Fate-mapping analysis utilizing single-cell RNA sequencing data from both mice and humans reveals that the macrophage population firstly emerging in the YS and FL typically expresses phosphatidylserine receptor T cell immunoglobulin and mucin domain 4 (TIMD4), and/or lymphatic endothelial hyaluronic acid receptor 1 (LYVE1), and/or folate receptor beta (FOLR2), which is thus designated as TLF^+^ macrophage. TLF^+^ macrophage exhibits high transcript conservation between mice and humans, suggesting TLF^+^ macrophage may be the intermediate subset during the evolutionary transition from MOs to KTRMs (15).

Most KTRMs are located in the renal medulla and distributed among the peritubular capillary endothelial cells, the tubule basement membrane, and Bowman’s capsule, facilitating the uptake of immune complexes (ICs) transported from the endothelial cell (16, 17). Alex Yashchenko et al. have found a macrophage subpopulation of CC Motif Chemokine Receptor 2 (Ccr2)^+^ KTRM in the renal cortex utilizing the single-cell RNA sequencing (scRNAseq), fate mapping, and parabiosis. The accumulation of Ccr2^+^ KTRM in the renal cortex under the regulation of CX3C chemokine receptor 1 (CX3cr1) can promote the progression of cortical-based cystic kidney disease (18).

KTRM is a crucial immune cell population in the kidney and play a significant role in the maintenance of renal homeostasis. CX3CL1 is a niche signaling molecule released from the kidney to recruit monocytes following the clearance of KTRMs niche. CX3cr1 is the sole known receptor for CX3CL1, which is specifically expressed in TRMs in the kidney, brain, and other organs (19). Under steady-state conditions in the renal environment, KTRMs constitute the largest proportions of renal leukocytes. They help to maintain the renal homeostasis by undergoing *in situ* proliferation and functioning through the CX3cr1/CX3CL1 axis. However, when KTRMs are depleted under pathological conditions, the KTRM pool can be reconstituted by bone marrow-derived monocytes, macrophages, and dendritic cell precursors (20–22). Mononuclear macrophages derived from monocyte-derived precursors (MDP) exhibit high expression of lysozyme (Lysm), while the expression of Lysm is downregulated in the differentiated, mature and long-term surviving KTRMs. Therefore, Lysm can be utilized to differentiate between newly-formed KTRMs derived from bone marrow precursor cells and the long-term surviving KTRMs. The expression of interferon regulatory factor 4 (Irf4) is essential for the differentiation of macrophage across all tissues induced by granulocyte-macrophage colony-stimulating factor (GM-CSF). The differentiation of bone marrow-derived TRMs into KTRMs is dependent on the fatty acid metabolism pathway mediated by fatty acid-binding protein 5 (FABP5) (23). Furthermore, interleukin 4 (IL-4), a key cytokine for TH2 response, can also promote the self-renewal and accumulation of TRMs (24). Taken together, the origin and location of KTRMs are tissue-specific and regulated through complicated mechanisms.

### 2.2 Phenotypic characteristics of KTRMs

F4/80 and CD11b are commonly used markers to identify the monocyte-macrophage system in mice. Within the kidney, BMRMs typically exhibit high expression of CD11b and low expression of F4/80. In contrast, KTRMs represent a distinct subpopulation characterized by low expression of CD11b and high expression of F4/80 (5). Additionally, surface markers such as CD11c can further categorize the mononuclear phagocytes (MPCs) resident in mouse kidney tissue into five discrete subgroups: CD11b^high^CD11c^high^ (MPC1), CD11b^high^CD11c^low^ (MPC2), CD11b^int^CD11c^int^ (MPC3), CD11b^low^CD11c^high^ (MPC4), and CD11b^-^CD11c^int^ (MPC5). Each of these subgroups exhibits distinct lineages and functions; for instance, MPC1 may play a role in initiating or regulating immune responses, while MPC2 demonstrates the highest phagocytic ability. MPC3 is the most abundant subgroup and is capable of producing high levels of the anti-inflammatory cytokine IL-10. MPC4 primarily participates in antigen presentation and the regulation of T-cell response, whereas MPC5 expresses transcription factors associated with dendritic cell development and immune regulation (25). Moreover, scRNA-seq analysis reveals that KTRMs in both rats and humans express the key surface markers CD74 and CD81 (26). This finding further validates the reliability of CD74 and CD81 as specific markers for identifying and confirming the presence of human KTRMs, providing a critical foundation for in-depth investigation into their functional characteristics. Notably, Stusies have shown that during mouse kidney development, KTRMs lack histocompatibility complex class II (MHC II) expression before postnatal day 7 (P7) and are even negative for MHC II before embryonic day 14.5 (E14.5). They mature between P7 and P14, during which the proportion of KTRMs expressing MHC II significantly increases (20). Therefore, high expression of MHC II can serve as a marker for the maturity of KTRMs. Alongside MHC II expression, the expression of T cell immunoglobulin and mucin domain 4 (TIM-4) and the MER receptor tyrosine kinase (MERTK) in KTRMs also transfroms from positive to negative (27). FcγR-mediated phagocytosis is one of the most direct and effective mechanisms of immune clearance within the immune system. It has been revealed that all subtypes of Fcγ receptors (FcγRs) are expressed in KTRMs, with FcγRIV being the most highly expressed subtype (16). KTRMs exhibit up-regulated expression of MerTK, FcγRI, FcγRI/II, and FcγRIV under pathological conditions, such as acute kidney injury (AKI) (20). These findings have suggested specific surface molecules can be utilized to differentiate between BMRMs and KTRMs. Furthermore, the dysregulated expression of MHC II and the activation of FcγRs suggest that KTRMs possess immune regulatory functions, including the uptake of immune complexes and antigen presentation. Notably, scRNA-Seq transcriptomic profiling reveals that KTRMs highly express genes associated with matrix protein synthesis, such as Col4a1, Col4a2, Lama3, and Lamb2, which are critical for the maintenance of glomerular basement membranes and interstitial structures. In contrast, BMDMs predominantly express pro-inflammatory genes, including Cxcl9 and Nos2 (28). Moreover, KTRMs exhibit a distinct gene expression pattern characterized by MRC1^high^HLA-DR^high^, distinguishing them from TRMs derived from other organs or BMDMs (29).

In the early stages of kidney development, KTRMs arrive at the kidney alongside newly formed blood vessels. They play a crucial role in clearing the misplaced glomerular progenitor cells through pinocytosis, which is essential for normal kidney development. Additionally, KTRMs produce angiogenesis-related molecules while spatially establishing connections with the renal blood vessels (30). This close interaction involves the phagocytic functions of vascular endothelial cells and intravascular blood cells, promoting the formation of connections among vascular endothelial cells and facilitating the establishment of a distinct vascular network in the kidney.

Colony-stimulating factor 1 (CSF-1) is the primary cytokine regulating the proliferation and differentiation of KTRMs by binding to the cell membrane receptor CSF1R. KTRMs stimulate the branching morphology of the ureteral bud and nephron formation through the CSF-1/CSF1R signaling pathway, thereby promoting kidney development (31, 32). In the homeostasis of mature kidneys, KTRMs are primarily responsible for monitoring the microvascular environment by regulating specific mediators, particularly the proteins and particles ranging from 20 to 700 kDa, or 10 to 200 nm, that enter the renal interstitium through vesicular transendothelial transport. Consequently, KTRMs can promptly initiate immune responses against infectious particles or immune complexes that enter the kidneys (17). Furthermore, compared to BMRMs, KTRMs exhibit a greater capacity for energy production through glycolysis and the mitochondrial oxidative respiratory chain, enhancing their ability to clear immune complexes and increasing the sensitivity to immune attacks (19). In summary, KTRMs are not only integral to the kidney development, but perform immune surveillance and maintain the homeostasis of the kidney microenvironment.

### 2.3 Pathophysiological roles of KTRM in renal injury-repair continuum

#### 2.3.1 Pathological activation of KTRM in AKI subtypes

AKI is a syndrome characterized by a rapid loss of kidney function. AKI is often attributed to extrarenal factors and is commonly classified based on its underlying causes (33, 34). For instance, ischemia-reperfusion injury (IRI) results from an acute interruption or significant reduction of blood flow in the renal vessels. Obstructive AKI is caused by urinary tract obstruction. Nephrotoxic AKI arises from exposure to nephrotoxic substances, while infection-related AKI is induced by systemic or local infections (35–38). A primary factor that exacerbates tissue damage in AKI is the macrophage-mediated inflammatory response (39, 40). Upon the onset of AKI, KTRMs are the first cells activated in response to signals indicating kidney injury, usually leading to changes to their spatial positioning. Concurrently, these macrophages enhance the inflammatory response by adopting a pro-inflammatory phenotype, thereby responding to triggers of renal injuries. The roles of KTRMs in different types of AKI will be discussed in details as follows.

A previous study has identified seven distinct subpopulations of KTRMs located in various nephron-enriched regions through single cell RNA sequence (scRNAseq) and spatial transcriptomic analysis of KTRMs from both quiescent (uninjured) mice and those subjected to bilateral ischemia-reperfusion injury (BIRI) (41). The transcriptomic characteristics of these KTRM subgroups suggest their involvement in several functions, such as the type I interferon response, heme/iron metabolism, inflammation, and antibacterial responses. Following acute ischemic injury, the transcriptional programming and spatial distribution of each KTRM subgroup undergo significant changes. For instance, one subpopulation of KTRMs originally located in the medulla relocates to the cortical area after injury. Additionally, certain subpopulations express inflammation-regulating genes, such as CXCL2, CCL3, and CCL4, as well as fibrosis-related genes like Pf4 and CD206 (41). Notably, these alterations persist for at least 28 days, suggesting that during AKI, KTRMs respond to renal tissue damage through transcriptomic reprogramming and changes in localization. Furthermore, RNA velocity analysis using Monocle2 reveals that the expression of repair-associated genes, such as Vegfa, is upregulated in Mertk^+^ KTRMs in the early stages of injury, thereby promoting the angiogenesis and tissue repair (42). This shift enables KTRMs to restore the homeostasis by regulating extracellular matrix (ECM) remodeling and fibrosis.

KTRMs play a crucial role in combating kidney infections caused by *Candida*. KTRMs can internalize *Candida* conidia through phagocytosis and employ endogenous killing mechanisms (43). Additionally, KTRMs secrete Cxcl2 in an autophagy-dependent manner, recruit neutrophils, and perform essential functions related to anti-infection and immune surveillance (43). In 2021, Yi Juan Teo et al. found a subpopulation of CD169^+^ KTRMs that enhanced host resistance by promoting the release of IFN-γ and increasing reactive oxygen species (ROS) in neutrophils during acute systemic candidiasis (44). Furthermore, the antifungal activity of KTRMs is regulated by integrin αXβ2, which not only contributes to their anti-infectious effects but also helps prevent kidney tissue damage associated with *Candida* infection (45).

TNF-α is the primary pro-inflammatory cytokine implicated in the progression of various diseases. It can induce renal damage associated with sepsis and exacerbate podocyte injury, leading to a decline in renal function (46). It has been well demonstrated that in a purulent AKI mouse model induced by cecal ligation and puncture (CLP), both Ly6C^+^ monocyte-derived macrophages and Ly6C^-^KTRMs exhibit characteristics of M1 polarization, as evidenced by high expression levels of CD80 (47). Consequently, during the early stages of inflammation, the activated KTRMs can produce substantial amounts of ROS and TNF-α, which activate monocyte-derived macrophages to combat pathogenic bacterial infections. However, the abandunt inflammatory response also significantly contributes to septic AKI. This study found that CRP peptide can bind to FcγRI on the surface of KTRMs, thereby regulating the excessive activation of these macrophages during late inflammation, which decreases levels of ROS and TNF-α, ultimately mitigating kidney damage (47). Furthermore, long-term exposure to PM2.5 significantly promotes the release of TNF-α via the inactive rhomboid protein 2 (iRhom2)/TACE/TNF-α signaling axis in KTRMs, contributing to podocyte damage and the deterioration of renal function and thereby exacerbating the progression of renal injury (48).

Heat stress-related AKI is associated with reduced levels of carnitine and damage to the fatty acid oxidation (FAO) pathway. In this type of kidney injury, there is not only a shift of KTRMs to the M1 pro-inflammatory phenotype but also a reduction in the overall number of KTRMs, which contributes to the progression of renal injury and ultimately renal fibrosis (49). However, the application of L-carnitine can prevent kidney damage by enhancing the FAO pathway and mitigating the pro-inflammatory polarization of KTRMs.

In summary, these studies have demonstrated that KTRMs and BMRMs exhibit synergistic pro-inflammatory effects in response to various types of kidney injury. Additionally, KTRMs can be activated through multiple pathways, exacerbating the inflammatory response that lead to kidney tissue damages. Targeted inhibition of KTRM activation facilitates a phenotypic transition to M2 macrophage phenotype, promoting the kidneys’ recovery and aiding in the transition to a post-injury repair state. This approach may represent a promising strategy for the treatment of kidney injury.

#### 2.3.2 Mechanisms of KTRMs during post-AKI tissue restoration

Renal tubular epithelial injury resulting from macrophage-mediated immune activation and inflammatory response is the primary manifestation of AKI. Macrophages exhibit both pro-inflammatory (M1, classical activation) and tissue repair (M2, alternative activation) phenotypes during AKI, suggesting their bidirectional effects (50). As key players in the intricate processes of kidney injury and repair, KTRMs demonstrate both pathogenic and protective roles. KTRMs are essential for maintaining kidney homeostasis. Their functions are influenced by phenotypic transformation, which is driven by various cytokines and activation pathways. This transformation stimulates the proliferation of renal tubular epithelial cells, mitigates renal inflammatory responses, and facilitates the overall repair process in AKI.

Colony-stimulating factor-1 (CSF-1), produced by the renal proximal tubule, plays a crucial role in mediating the repair of AKI. As the extent of cell necrosis and apoptosis in the damaged kidney increases, CSF-1 promotes the proliferation of KTRMs and dendritic cells, facilitating a phenotypic transition from M1 (pro-inflammatory) to M2 (tissue repair) (50–52). Concurrently, the proliferative activity of renal tubular cells increases. These actions collectively inhibit the spread of inflammation, support the progression of renal injury into the repair stage, and may even contribute to renal interstitial fibrosis in the later stages of AKI (50–52). Furthermore, studies have confirmed that CSF-1 exerts a nutritional effect on the development of kidney embryos, which is also involved in tissue repair following kidney injury (32).

In 2019, Jeremie M. Lever and the colleagues have found that KTRMs undergo transcriptional reprogramming to the MHCII^–^KTRMs developmental phenotype following AKI (20). This reprogramming is beneficial for mitigating inflammation and promoting kidney damage reduction and tissue healing. In the context of renal injury induced by cisplatin, KTRMs respond to injury signals by upregulating the PGE2 receptors Ptger2 (EP2) and Ptger4 (EP4) (27). This response is associated with the downregulation of MHCII expression through the prostaglandin (PG) signaling, which reduces renal inflammation and prevents excessive immune activation (27). Furthermore, KTRMs can facilitate kidney repair following injury by upregulating the ligand Wnt4, the receptor Fzd1, and the co-receptor Lrp6, thereby activating the Wnt/β-catenin signaling pathway (20). In ischemia-reperfusion injury-associated AKI (IRI-AKI), CD169^+^ KTRMs directly interact with renal blood vessels, leading to reduced expression of intercellular adhesion molecule-1 (ICAM-1) on the vascular endothelial cells. This interaction helps prevent excessive inflammatory responses by inhibiting neutrophil accumulation (53). ScRNAseq data from previous studies have implicated that KTRMs are enriched in pathways associated with classical macrophages, such as antigen processing and presentation (54). Accordingly, KTRMs exhibit functional synergy with BMRMs. Besides, some other research has demonstrated that KTRMs and BMRMs play complementary roles during IRI, particularly in the acute phase of AKI (55). In the early stages of renal injury, KTRMs rapidly respond to local damage, regulating inflammation through phagocytosis and the production of anti-inflammatory factors. In contrast, BMRMs are crucial for initiating and amplifying the inflammatory response. In the later stages of renal injury, KTRMs contribute to the stability of renal microenvironment and promote the migration and differentiation of myeloid cells. Additionally, BMRMs can transform into the repair phenotype, facilitating tissue repair by generating growth factors and enhancing cell proliferation (55). This process is vital for balancing kidney inflammation and repair mechanisms, thus maintaining kidney function and promoting recovery after injury. Taken together, KTRMs can synergize with classical macrophages to respond to renal injury and facilitate tissue repair alongside BMRMs. However, this interaction may be influenced by immune dysregulation following injury, which is associated with an increased risk of chronic kidney diseases. In addition, the altering effects of BMRMs and KTRMs on renal injury and repair have been well documented previously. The transcriptomic profiling and flow cytometric analyses have highlighted KTRMs displayed markedly higher expression of the inhibitory immune checkpoint molecule V-domain Ig suppressor of T-cell activation (VISTA) compared to BMDMs in the IRI mouse model, and the VISTA^+^KTRMs are shown to play a pivotal role in accelerating tissue repair by the efficient clearance of apoptotic cells and robust inhibition of T cell activation and proliferation in IRI (56). Nonetheless, BMDMs are demonstrated to predominantly function in the early stage of inflammatory response via upregulating the expression of inflammation-related genes, such as Ly6c2 and Ccr2, while BMDMs possess relatively limited capacity for tissue repair (56). These findings have underscored the different roles of BMRMs and KTRMs in regulating renal injury and repair

In a renal injury model of unilateral ureteral obstruction (UUO), researchers observed that the apoptotic KTRMs produce milk fat globule epidermal growth factor 8 (MFG-E8). It regulates the reprogramming of anti-inflammatory macrophages and promotes the accumulation of CD206^+^ M2 macrophages, which inhibits the activation of the NLRP3 inflammasome and the release of pro-inflammatory cytokines, ultimately alleviating kidney tissue damage (57). KTRMs are also activated in kidney allografts, where they mediate acute rejection and contribute to kidney injury (58, 59). However, following cold ischemia (CI)-induced renal transplantation-related AKI and reversible IRI, F4/80^+^ CD11c^+^ KTRMs upregulate the expression of the surface molecule IL-1R8, thereby inhibiting TLR4 activation and prompting KTRMs to transform to M2-type macropages. KTRMs play a crucial role in preventing graft fibrosis and abnormal humoral immune response by maintaining the balance between pro-inflammatory and anti-inflammatory/reparative macrophage responses (60). In heat stress-related AKI, heat acclimation (HA) can induce the expression of heat shock protein 70 (HSP 70) in KTRMs. HSP 70 plays a critical role in inhibiting the apoptosis of KTRMs, promoting the repair of renal tubular damage, and reducing the severity of AKI (61). This underscores the potential of KTRMs as a promising target for therapeutic interventions in heat stress-related AKI.

All these findings have strongly support that KTRMs possess significant tissue repair capabilities as a resident cell population in the kidney. Following kidney injury, the activation of KTRMs facilitates the transition of kidney tissue into a repair state. This process is primarily linked to the reprogramming of KTRMs into the M2 anti-inflammatory phenotype and their interaction with BMRMs.

### 2.4 Crosstalk between KTRMs and other cells in renal networks

#### 2.4.1 Interaction of KTRMs with renal parenchymal cells

**Figure 1:**
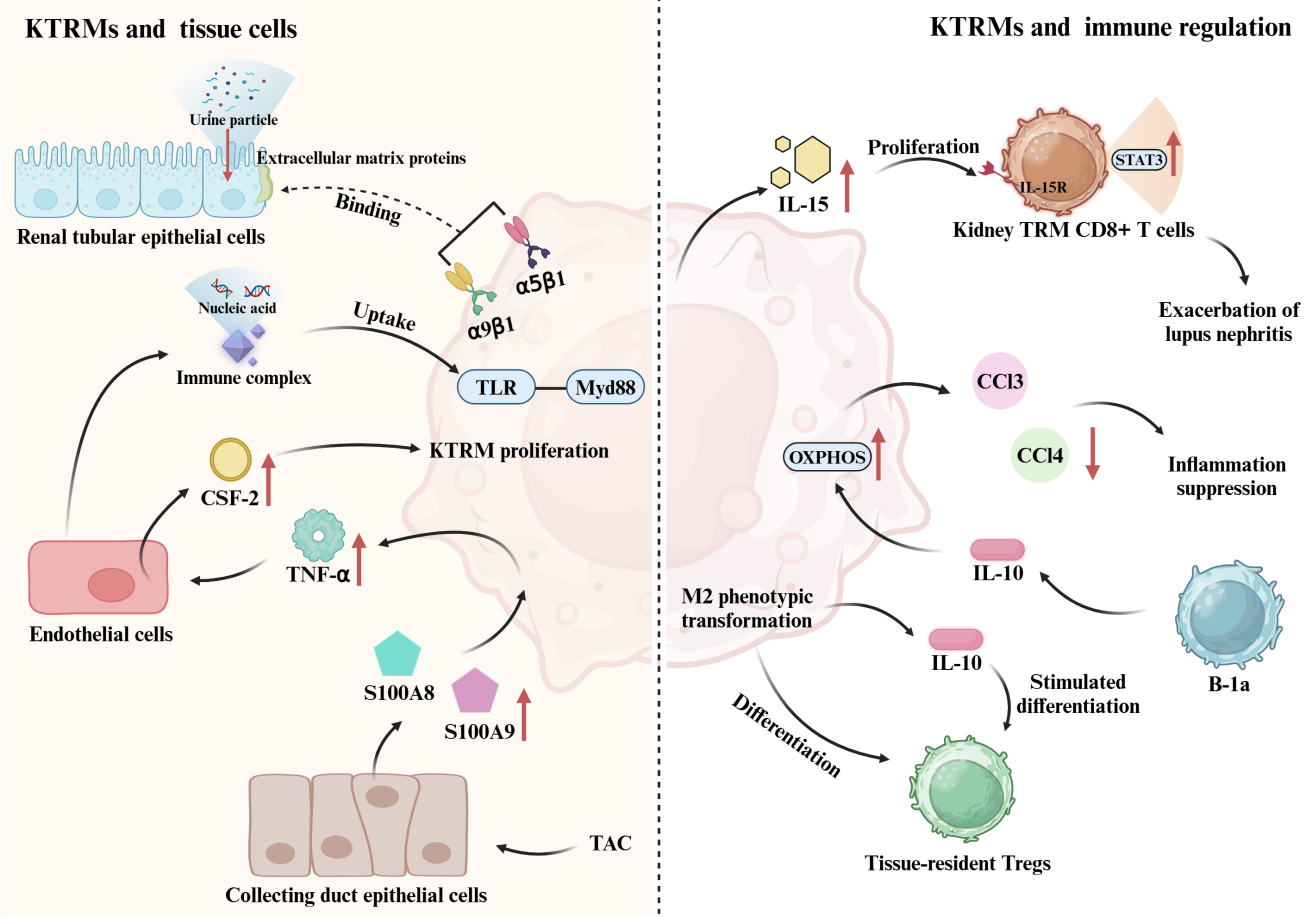
Crosstalk between KTRM and renal cellular networks. KTRMs bind to extracellular matrix proteins of tubular epithelial cells via integrin heterodimers α9β1 and α5β1, monitoring and clearing urinary particles. They also establish contacts with peritubular endothelial cells to monitor and phagocytose circulating immune complexes; when these complexes contain nucleic acids, KTRMs can be activated via the TLR-Myd88 pathway. Additionally, in heart failure, collecting duct epithelial cells induced by transverse aortic constriction (TAC) express S100A8 and S100A9, which promote KTRM activation to produce TNF-α. TNF-α induces renal endothelial cells to generate CSF2, promoting KTRM proliferation. This cardiorenal regulatory network helps maintain homeostasis. KTRMs can also establish connections with other immune cells in the kidney. KTRMs produce IL-15, which activates the IL-15/IL-15R/STAT3 signaling pathway in tissue-resident memory CD8+ T cells, inducing their proliferation and activation, thereby exacerbating lupus nephritis (LN). Tissue-resident Tregs regulate the M2 phenotypic shift of KTRMs to prevent inflammation spread and promote repair. KTRMs also secrete IL-10 to stimulate the differentiation of regulatory T cells. Moreover, the predominant B-1a subpopulation of kidney tissue-resident B cells increases the expression of OXPHOS pathway genes in KTRMs via IL-10 production, promoting their transition to an M2 anti-inflammatory phenotype and inhibiting the production of monocyte-recruiting chemokines (CCL2, CCL3, and CCL4) by KTRMs.

During the establishment of the immune landscape of human kidney, tissue-resident myeloid and lymphoid immune cell networks acquire anti-infection-related transcriptional programs after birth. Notably, the interactions between the epithelial cells and immune cells orchestrate the localization of KTRMs in areas of the kidney that are particularly vulnerable (62). This localization enables KTRMs to perform essential functions related to homeostasis maintenance and defense against infections. KTRMs located in the renal medulla interact with the extracellular matrix proteins of renal tubular epithelial cell (TEC) through integrin heterodimers α9β1 and α5β1, forming transepithelial protrusions that extend deep into the renal tubule lumen. This interaction allows for the monitoring and removal of urine particles, thereby preventing tubular obstruction and the formation of kidney stones (63, 64). Additionally, KTRMs are localized on the luminal side of peritubular capillaries, allowing them to create a kidney-specific anatomical and functional unit with peritubular endothelial cells. This interaction is crucial for initiating the inflammatory response triggered by small immune complexes in the kidney. The structure relationship between KTRMs and endothelial cells enables KTRMs to monitor and phagocytose circulating immune complexes that cross the endothelial barrier into the renal interstitium (17). Simultaneously, they activate FcgRIV-dependent inflammatory responses and recruit monocytes and neutrophils. Furthermore, when immune complexes contain nucleic acids, the FcgRIV-dependent cellular immune response can synergistically activate KTRMs through the TLR-Myd88 pathway (17). In heart failure, the dynamic coordination between the heart and kidneys plays a crucial role in the heart’s adaptive response to pressure stress. CSF2 is a key cytokine produced through intercellular interactions among the activated collecting duct (CD) epithelial cells, tissue macrophages, and endothelial cells within the kidney (65). Renal CD cells exhibit elevated expression of S100A8 and S100A9 in response to transverse aortic constriction (TAC), which promotes the activation of KTRMs to produce TNF-α. In turn, TNF-α stimulates renal endothelial to produce CSF2. The interaction between KTRMs and renal interstitial cells contributes to the establishment of a heart-kidney regulatory network that supports adaptive immunity in response to cardiac stress (65). This suggests that KTRMs can exert immune functions by forming specific connections with kidney tissue cells, thereby regulating immunity and maintaining the homeostasis in kidneys (**Figure 1**).

#### 2.4.2 Interaction of KTRMs with other renal immune cells

In addition to establishing connections with kidney tissue cells to jointly maintain kidney homeostasis, KTRMs also interact with other immune cells to regulate the kidney microenvironment. Studies have confirmed that TRMs possess immunomodulatory effects. For instance, naive tissue-resident large-cavity macrophages (LCMs) can be modulated by type 2 helper T cells (Th2) in pleurisy induced by nematode infection (66). Th2 cells promote the migration of monocytes to LCMs by releasing Th2-type cytokines, particularly IL-13 and IL-4 (66). This immunomodulatory function of KTRMs is further supported by evidence from various kidney diseases.

Activated T lymphocytes play a crucial role in tissue damage associated with nephrotoxic nephritis. KTRMs can recruit the activated CD4^+^ T cells in the kidney, which is linked to the progression of crescentic nephritis (67). The severity of lupus nephritis (LN), a common complication of systemic lupus erythematosus (SLE), is closely associated with tissue-resident memory CD8^+^ T cells. Interleukin-15 (IL-15) produced by KTRMs induces the proliferation and activation of tissue-resident memory CD8^+^ T cells via activating the IL-15/IL-15R/STAT3 signaling pathway (68). However, the interaction between KTRMs and tissue-resident memory CD8^+^ T cells can be inhibited by Zhen-Wu-Tang (ZWT) (68). ZWT not only suppresses the expression of IL-15 in KTRMs but also reduces the phosphorylation of STAT3 and CD122 (IL-2/IL-15Rβ) in tissue-resident memory CD8^+^ T cells. This inhibition blocks the activation of the JAK/STAT signaling pathway, thereby suppressing the proliferation and activation of both KTRMs and tissue-resident memory CD8^+^ T cells, ultimately preventing the progression of LN (68).

In mice with AKI, tissue-resident regulatory T cells (Tregs) exhibit distinct transcriptional characteristics at various stages. During the fibrosis stage of renal injury, Tregs primarily upregulate the expression of genes associated with inflammation and apoptosis. In contrast, there is an upregulation of angiogenesis-related genes during the repair stage. Throughout the repair process in AKI, tissue-resident Tregs play a crucial role in preventing the spread of inflammation and promoting tissue repair by facilitating the transition of KTRMs to the M2 phenotype. Moreover, some studies have indicated that KTRMs can secrete the anti-inflammatory cytokine IL-10, which stimulates the differentiation of regulatory T cells and coordinates the recruitment of inflammatory cells in the context of renal injury (25, 27).

B-1a cells represent the primary subpopulation of tissue-resident B cells and play a crucial role in regulating the metabolic and functional status of KTRMs through the production of IL-10 (69). IL-10 induces the transformation of KTRMs into an M2 anti-inflammatory phenotype. By enhancing the expression of genes associated with the oxidative phosphorylation (OXPHOS), B-1a cells inhibit both the phagocytosis and the production of monocyte-recruiting chemokines by KTRMs, specifically CCL2, CCL3, and CCL4 (69).

In summary, the renal microenvironment features a distinct connection between KTRMs and renal interstitial cells, as well as a reciprocal regulatory interaction between KTRMs and various types of immune cells within the renal immune cell pool. This intricate interaction mediates the recruitment and development of inflammatory cells, leading to phenotypic changes in KTRMs that ultimately influence the progression of kidney disease (**Figure 1**).

## 3 Functional mechanisms of KTRMs in renal pathology

### 3.1 KTRMs in immune nephritis: regulation of inflammation and autoantibodies production

Studies have shown that KTRMs are located between the capillary endothelium, the canalicular basement membrane, and Bowman’s capsule. They mediate the uptake of immune complexes through FcγRIV, triggering macrophage activation, the production of inflammatory factors, such as TNF-α, and the recruitment of infiltrating monocytes and neutrophils (70). In the MRL-Lpr SLE mouse model, a significant increased proportion of KTRMs is observed in lupus nephritis (LN) tissues, with a notable distribution in the renal cortex and clear evidence of glomerular encapsulation (71). KTRMs not only secrete monocyte chemotactic factors, CCL4 and CCL8, to promote the recruitment of renal leukocytes but also produce B cell tissue niche factors, such as BAFF. This supports the formation of tertiary lymphoid structures containing B/plasma cell aggregates that produce local autoantibodies (71). These findings have implicated that in LN, KTRMs primarily play a role in coordinating the recruitment of inflammatory cells.

It has been found that mesenchymal stem cells (MSCs) can regulate the renal immune microenvironment and increase the proportion of KTRMs (72). When applying MSCs treatment, new KTRMs can be derived from pro-inflammatory monocytes and macrophages, but exerting significant anti-inflammatory effects (72). This reveals that MSC has a considerable regulatory effect on KTRMs and effectively inhibits the progression of LN. Furthermore, nuclear factor-κB (NF-κB) has been identified as a crucial inflammatory transcription factor in KTRMs (73). The activation of NF-κB signaling plays a pivotal role in the immune complex-mediated renal inflammation and glomerulonephritis. Additionally, in a tyrosine phosphatase-1 (Shp1)-CKO mouse model of autoimmune nephritis, it has been demonstrated that Shp1, as a negative regulator of the M-CSF signaling pathway, can inhibit the proliferation and activation of KTRMs, thereby suppressing autoimmunity and the development of nephritis (74). All these findings have suggested that KTRMs can exacerbate renal tissue damage in LN through inflammatory activation pathways. Consequently, targeting KTRMs is a promising therapeutic strategy for managing immunoinflammatory nephropathy.

### 3.2 KTRMs in diabetic nephropathy: metabolic dysregulation and inflammatory cascades response

The progression of diabetic nephropathy (DN) is characterized by immune cell-mediated renal inflammation and fibrosis. A significant contributor to the worsening of DN is the activation of Toll-like receptor 9 (TLR9) and the downregulation of superoxide dismutase 2 (SOD2), which leads to mitochondrial dysfunction. In a high-glucose environment, KTRMs establish a detrimental feedback loop with renal tubular epithelial cells and other immune cells. Under the influence of advanced glycation end products (AGEs), KTRMs release damage-associated molecular patterns (DMAPs), resulting in complement activation and the release of inflammation-related mediators, such as IL-1β, IL-6, and IL-10 (75, 76). Besides, the high glucose environment prompts renal tubular epithelial cells to upregulate the expression of CCL2 and M-CSF1, leading to KTRM dysfunction and the activation of immune cells and thereby accelerating the progression of DN (75, 76). In 2022, Seigo Ito et al. have discovered that in obesity-induced DN mice (db/db), KTRMs exhibit elevated expression of TLR9 (77). Furthermore, increased levels of cytoplasmic mitochondrial DNA (mtDNA) can enhance the production of mitochondrial reactive oxygen species (mtROS) and the expression of TNF-α in KTRMs through the activation of the TLR9-MyD88 signaling pathway, thereby exacerbating the inflammatory response and tissue damage in DN (77). However, the application of L-carnitine has been shown to effectively enhance mitochondrial function, mitigate renal tubular damage, and downregulate TLR9 expression in KTRMs, thereby reducing the severity of DN (77). The scRNA-seq analyses of renal immune cells in both normal and OVE26 diabetic mice have demonstrated that KTRMs in normal mice were primarily characterized by TREM2^high^ and Mrc1^high^ subpopulations, which mediated tissue repair and anti-inflammatory responses, respectively (78). Conversely, in OVE26 diabetic mice, there is a notable increase in the TREM2^high^ KTRMs alongside a reduction in the Mrc1^high^ KTRMs (78). These observations indicate that KTRMs undergo a dynamic phenotypic shift from an anti-inflammatory state to a pro-inflammatory state, suggesting a tissue-repair phenotype during the pathogenesis of DN. This phenotypic plasticity may represent a critical mechanism underlying the repair process in DN. These studies have confirmed that KTRMs play critical roles in the regulation of DN. The activation of KTRMs is essential for the recruitment of inflammatory cells to renal tissues and for addressing the specific needs of the renal microenvironment under pathological conditions. This area of research is currently highly active, highlighting the importance of KTRMs in renal inflammation and their potential as therapeutic targets.

### 3.3 Dual roles and targeting potentials of KTRMs in renal fibrosis

**Figure 2:**
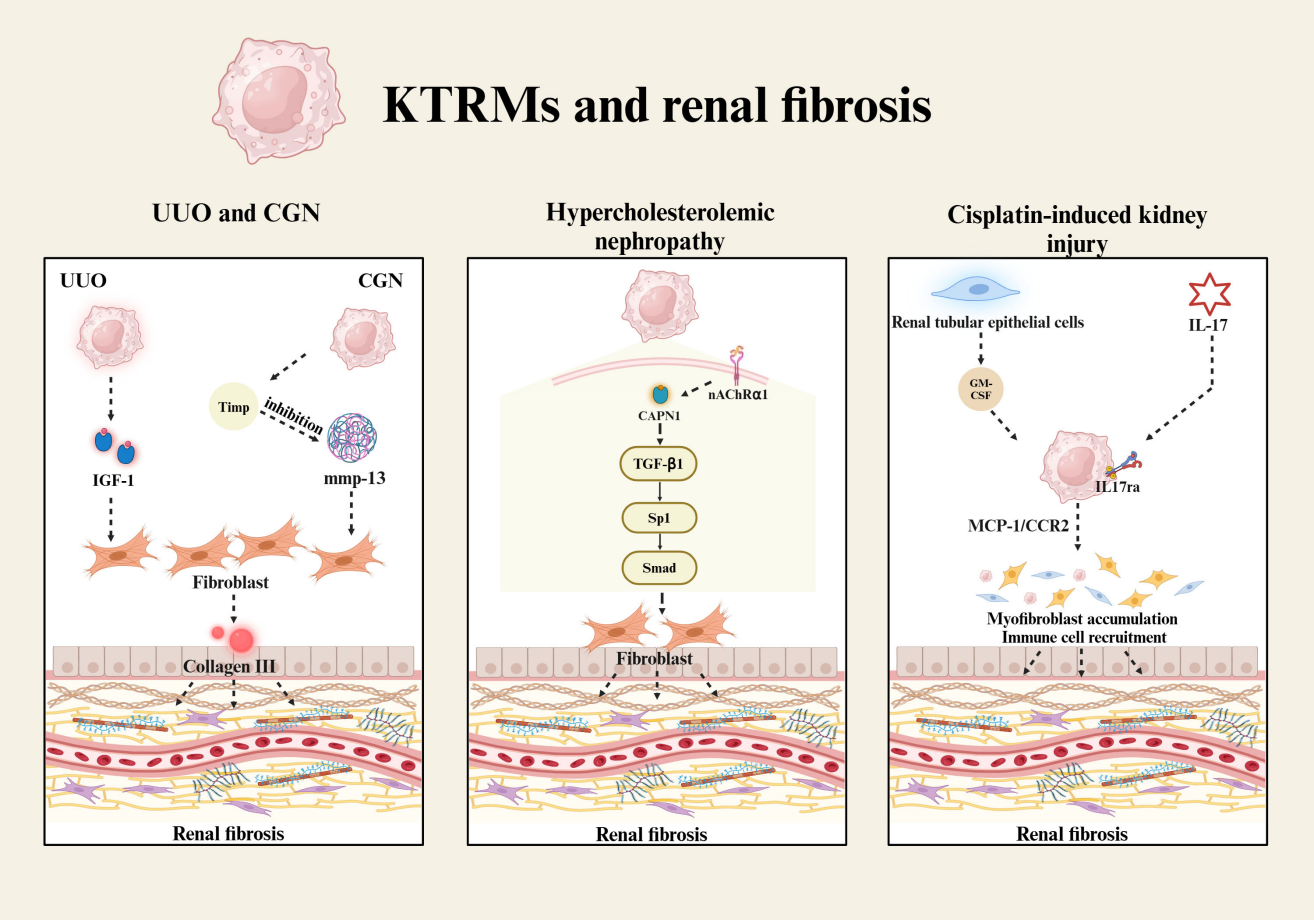
KTRMs and renal fibrosis. The development of renal fibrosis is associated with KTRMs that induce the proliferation and activation of fibroblasts. In UUO, KTRMs regulate renal fibrosis by secreting IGF-1. In CGN, KTRMs promot fibroblast proliferation by releasing tissue inhibitors of Timp to inhibit mmp-13 activity. In hypercholesterolemic nephropathy, calpain-1/nAChRα1 in KTRMs activates the TGF-β1/Sp1/Smad signaling pathway to induce fibroblast activation. In cisplatin-mediated renal injury, renal tubular epithelial cells secrete GM-CSF, which activates KTRMs, leading to the upregulation of MCP-1 and CCR2. Additionally, the activation of IL-17 signaling promotes the recruitment and activation of myofibroblasts and immune cells, ultimately contributing to the progression of renal fibrosis.

Tubulointerstitial fibrosis is the primary manifestation of renal fibrosis, characterized by the loss of renal tubular epithelium, along with the activation and accumulation of fibroblasts and α-smooth muscle actin (α-SMA)-positive interstitial myofibroblasts. This pathological process results in the deposition of extracellular matrix (ECM) proteins. Most myofibroblasts originate from the epithelial-mesenchymal transition (EMT) of renal tubular epithelial cells, with transforming growth factor-beta 1 (TGF-β1) serving as the principal regulatory factor that induces EMT. TGF-β1 mediates the downregulation of E-cadherin and the upregulation of α-SMA through the TGF-β1/Sp1/Smad signaling pathway (79). Additionally, NF-κB functions as a crucial transcription factor in the induction of tubulointerstitial fibrosis. Its activation promotes the expression of pro-inflammatory mediators, such as TNF-α, IL-1β and vascular cell adhesion molecule-1 (VCAM-1) (79). In the unilateral ureteral obstruction (UUO) renal fibrosis model, three macrophage populations have been identified in kidney tissues, all originating from the Ly6C^high^ bone marrow-derived monocytes (80). Among these, Ly6C^low^-KTRMs exhibit a pro-fibrotic M2-like phenotype. These KTRMs are capable of secreting cytokines, such as IGF-1, which promote the progression of fibrosis through the paracrine signaling to fibroblasts (80). Research on progressive kidney injury in mice with crescentic glomerulonephritis (CGN) has suggested KTRMs play an important role in regulating the proliferation and apoptosis of renal myofibroblasts (67). They inhibit the activity of collagenase MMP-13 by regulating the expression of tissue inhibitors of metalloproteinases (TIMPs), thereby maintaining a high turnover state of renal myofibroblasts and collagen III deposition (67) (**Figure 2**). This activity contributes to an increase in both the quantity of glomerular crescents and the extent of scarring.

In hypercholesterolemic nephropathy, KTRMs are the primary cell type expressing the nicotinic acetylcholine receptor α1 (nAChRα1). As an upstream protein, nAChRα1 regulates the expression and activation of calpain-1, subsequently facilitating the TGF-β1/Sp1/Smad signaling pathway (81). The signal axis contributes to the loss of integrity in both glomerular and tubular basement membranes, as well as an increase in α-smooth muscle cell (α-SMC)^+^ fibroblasts (81) (**Figure 2**). Kidney damage induced by low-dose cisplatin can easily progress to renal fibrosis in later stages. The study by Sophia M. Sears and the colleagues has reported that liposome-encapsulated clodronate effectively delays the progression of renal fibrosis by depleting KTRMs and CD206^+^ M2 macrophages, thereby reducing collagen deposition, myofibroblast accumulation, and the production of inflammatory cytokines (82). During kidney injury and repair, granulocyte-macrophage colony-stimulating factor (GM-CSF) is continuously expressed by renal tubular epithelial cells. GM-CSF significantly upregulates the expression of monocyte chemoattractant protein-1 (MCP-1) and its receptor CCR2 in renal macrophages (83). The activation of the GM-CSF/MCP-1/CCR2 signaling pathway prolongs the survival of renal macrophages, induces the accumulation of myofibroblasts, and promotes the development of renal fibrosis (83). Furthermore, interleukin 17 receptor A (IL-17RA) is involved in KTRMs-mediated renal fibrosis, which not only recruits the innate immune cells through the IL-17/IL-17RA signaling pathway but also influences the formation of tissue macrophages (84) (**Figure 2**). The activation of IL-17/IL-17RA signaling pathway in KTRMs contributes to the progression of renal inflammation and fibrosis.

In summary, all these findings support that KRTMs promote the progression of fibrosis by regulating the renal tissue homeostasis. KTRMs primarily mediate the accumulation and activation of fibroblasts via the paracrine signaling pathway, ultimately driving renal tissue toward fibrosis. Understanding the mechanisms to restore the homeostasis of KTRMs could provide valuable insights for the treatment of renal fibrosis.

### 3.4 KTRMs in shaping tumor immune microenvironment in renal carcinoma

Clear cell renal cell carcinoma (ccRCC) is a multicellular ecosystem, in which KTRMs significantly influence the composition of the immune microenvironment and contribute to the heterogeneity of ccRCC (85, 86). Dorothee Brech et al. have found a subpopulation of dendritic cells termed “enriched-in-renal cell carcinoma” (erc) DCs within ccRCC (87). Transcriptional profiling analysis classified ercDCs as KTRMs, revealing their co-expression of both M1 and M2 markers. This unique chimeric cell type exhibits characteristics associated with tissue remodeling and immune regulation. Further studies have shown that KTRMs play a significant regulatory role in the tumor microenvironment of ccRCC (88). They enhance the expression of novel immune checkpoint genes, HAVCR2 and LAG3, highlighting their involvement in tumor immune escape. Furthermore, the presence of exhausted CD8^+^ T cells in proximity to KTRMs suggests that these cells are actively engaged in modulating the immune microenvironment of ccRCC, thereby promoting its progression (89).

### 3.5 Thrombo-inflammatory transition of KTRMs in hemolytic uremic syndrome

Enterohemorrhagic Escherichia coli infection is the primary pathogen responsible for hemolytic uremic syndrome (HUS), with the Shiga toxin it produces leading to thrombotic microangiopathy in the kidneys (90). In a STC-hus mouse model, it has been observed that during the onset of HUS, KTRMs are activated and transformed to the M1-type macrophages. The upregulation of TNF-α and neutrophil chemotactic factors Cxcl1 and Cxcl2 in KTRMs not only contributes to damage in endothelial cells and platelets but also exacerbates the recruitment and activation of neutrophils. Consequently, the activation of KTRMs emerges as a key factor in the renal injury associated with HUS (91). These findings suggest that the activation of KTRMs intensifies the inflammatory response, leading to further damages to renal tissues.

### 3.6 Epithelial-mesenchymal signaling regulated by KTRMs in renal cysts

The study by Kurt A. Zimmerman et al. has demonstrated that KTRMs transitioned from CD11c^low^ to CD11c^high^ isoforms during kidney maturation post-birth in mice (92). However, In the context of renal injury, ciliary mutant mice, which lack cilia, exhibit a proliferation of juvenile-like CD11c^low^ isoform KTRMs (92). These cells accumulate in the vicinity of epithelial-derived renal cysts, resulting in the rapid formation of renal cysts. These manifestations of KTRMs are driven by the local paracrine and juxtacrine signaling pathway involving membrane-bound CSF1 (mbCSF1) and its receptor CSF1R in injured renal proximal tubule epithelial cells (92). Furthermore, in 2023, Randee Sedaka et al. reported that a high-protein diet resulted in the upregulation of SNAT3 and PEPCK in renal tubular epithelial cells, enhancing glutamine delivery to the kidneys and subsequently triggering renal hypertrophy (93). The injury-induced signals responsible for kidney hypertrophy are the primary factors in the proliferation and activation of KTRMs, which are further associated with the accumulation of KTRMs and the development of renal cysts (93). This indicates that while the progression of renal cysts is dependent on the activation of KTRMs, KTRMs themselves are not the initiating factor in the formation of these cysts.

In summary, KTRMs with different phenotypic characteristics play crucial roles in various types of kidney diseases (**Table 1**). In the early stages of the disease, KTRMs typically exhibit an M1 pro-inflammatory phenotype, characterized by the production of various inflammatory factors and chemokines that induce increased recruitment of inflammatory cells into kidneys. In contrast, during the later stages, KTRMs may undergo transcriptional reprogramming to an anti-inflammatory M2 phenotype, facilitating tissue repair and fibrosis in the kidneys. Moreover, the mutual regulation between KTRMs and other cell types in the kidney is also essential in the progression of kidney disease. Accordingly, the targeted regulation of KTRMs represents a promising avenue for immunotherapy in renal diseases and holds significant potential for future therapeutic research.

**Table 1:** Roles and mechanisms of KTRMs in different renal diseases.

Renal diseases	Roles (Protective/Detrimental)	Molecular mechanisms	Refs.
IRI	Protective Functions: Early Mertk^+^ KTRMs promote angiogenesis; later, they shift to M2 phenotype for ECM remodeling and tissue repair.	• Upregulation of Vegfa expression	(42)
	Damaging Functions: KTRMs spatial distribution abnormalities promote inflammation and fibrosis.	• Increased expression of inflammatory genes (CXCL2, CCL3, CCL4) and activation of fibrosis-related genes (Pf4, CD206)	(41)
Candida-Related AKI	Protective Functions: The CD169^+^ subpopulation enhances the host’s antifungal capacity.	• Promotion of IFN-γ release and ROS activity	(44)
Septic AKI	Damaging Functions: The Ly6C^+^ subpopulation undergoes M1 polarization, exacerbating inflammation.	• Release of ROS and TNF-α	(47)
Heat Stress-Related AKI	Damaging Functions: KTRMs shift to the M1 phenotype, decrease in number, and promote fibrosis progression.		(49)
UUO-induced kidney injury	Protective Functions: Promote M2 macrophage accumulation and alleviate renal inflammatory injury.	• Release of MFG-E8 to reduce NLRP3 inflammasome activation	(57)
Lupus nephritis	Damaging Functions: Coordinate the recruitment and maintenance of inflammatory cells in tertiary lymphoid follicles to produce local autoantibodies	• Secretion of CCL4, CCL8 and BAFF	(71)
Diabetic nephropathy	Damaging Functions: Form a malignant feedback loop with renal tubular epithelial cells and other immune cells.	• Hyperglycemic environment induced up-regulation of CCL2, M-CSF1 expression	(75)
		• Up-regulated AGEs/DMAPs/IL-1β, IL-6 and IL-10	(76)
	Increased inflammation and activation of immune cells	• Up-regulated mtDNA/TLR9-MyD88/mtROS and TNF-α	(77)
Renal fibrosis	Damaging Functions: Renal destruction and Fibroblast accumulation	• Up-regulated TGF-β1/Sp1/Smad	(81)
	Fibroblast accumulation and activation	• Up-regulated GM-CSF/MCP-1/CCR2	(83)
	Recruitment of innate immune cells	• Up-regulated IL-17/IL17ra	(84)
Clear cell renal cell carcinoma	Damaging Functions: Regulates the immune micro-environment of ccRCC.	• Up-regulated expression of HAVCR2 and LAG3 and exhausted CD8+T surround	(89)
Hemolytic uremic syndrome	Damaging Functions: Damage to endothelial cells and platelets; inflammatory cell activation recruitment	• Up-regulated expression of TNF-α, Cxcl1 and Cxcl2	(91)
Renal cyst	Damaging Functions: Neoplastic KTRMs proliferation activation and promotion of renal cysts	• Activation of mbCSF1/CSF1R pathway in renal tubular epithelial cells	(92)
	Renal hypertrophy	• Up-regulated expression of SNAT3 and PEPCK	(93)

## 4 Precision therapy targeting KTRMs

### 4.1 Depletion of KTRMs

In recent years, the treatment strategies targeting KTRMs have garnered significant attention due to their central role in AKI and fibrosis. Studies have shown that the application of CSF1 receptor inhibitor PLX3397 significantly reduced cisplatin-induced tubular injury and fibrosis through the targeted ablation of KTRMs (94). Notably, the use of CRISPR/Cas9 technology to knock out the FIRE sequence of the CSF1R gene in mice can achieve complete ablation of KTRMs, without significant developmental defects (95). However, the long-term depletion of KTRMs may disrupt renal immune surveillance, and its safety still requires systematic evaluation. Further studies have revealed that the aldosterone antagonist esaxerenone exerted inhibitory effects on KTRM proliferation and M1 polarization by blocking the glucocorticoid receptor (MR)/CSF1 pathway, demonstrating the unique therapeutic advantages in the model of aldosterone-induced kidney injury (96). In the field of transplant immunology, blocking the CCL8-CCR8 signaling axis of KTRMs using anti-CCL8 mAb and anti-CCR8 mAb, or directly depleting KTRMs can significantly suppress the early allograft inflammatory response and reduce T cell infiltration, providing new insights into improving the survival (97). These studies have collectively suggested the potential use of KTRMs depletion in treating different diseases, whereas the clinical application potentials require further in-depth exploration.

### 4.2 Phenotypic modulation of KTRMs

The dynamic regulation of KTRMs polarization is a critical factor determining the outcome of kidney injury. In inflammatory diseases such as LN, the inhibition of the NLRP3/IL-33/ST2/NF-κB signaling axis in KTRMs using Honokiol not only prevents the activation of KTRMs but significantly reduces the abnormal interactions with renal tubular epithelial cells, thereby achieving the protective effects (98). In the IRI regeneration model, M2-type KTRMs exhibit high expressions of tissue repair genes such as Arg1 and Retnla, and promote microenvironment remodeling by producing the anti-inflammatory factors, for instance, IL-10 and TGF-β (99). In contrast, the activation of IFN-β signaling pathway in KTRMs using IL-37b not only suppresses cyst formation but downregulates the pro-inflammatory factors such as CXCL1 and CXCL4, demonstrating the therapeutic potential of metabolic-immune interaction regulation in the autosomal dominant polycystic kidney disease (ADPKD) (100). In addition, some epigenetic regulatory approaches, such as LNA-anti-miR-150, can reduce the M1 polarization of KTRMs by inhibiting miR-150 and thereby upregulating SOCS1 expression, leading to decreased production of pro-inflammatory factors such as IFN-γ and IL-6, and reduced infiltration of bone marrow-derived macrophages in LN (101). These findings have suggested that precise modulation of KTRM polarization requires an integrated strategy encompassing signaling pathway intervention, metabolic reprogramming regulation, and epigenetic modifications.

### 4.3 Intervention of monocyte-macrophage migration by KTRMs

In the early stages of kidney injury, the inflammatory amplification loop established by KTRMs through the secretion of chemokines such as CXCL1 and CCL2 has become a research hotspot for intervention (55). It has been revealed the enhanced glycolysis and fatty acid oxidation metabolism not only promote the proliferation and differentiation of KTRMs, but accelerate the migration of monocyte-derived macrophages through the upregulation of CCL2 and CX3CR1 (102). In the IRI model, the depletion of the gut microbiota has been demonstrated to significantly inhibit monocyte migration into the kidney and ameliorate the renal inflammation via reducing the expressions of CX3CR1 and CCR2, thereby decreasing the production of chemokines such as MCP-1 and MIP-2α (103). Therefore, targeting KTRMs by the intervention of monocyte-macrophage migration may become a critical approach to different renal diseases.

## 5 Conclusions and future directions

Numerous studies have demonstrated that KTRMs frequently undergo transcriptional reprogramming during renal injury, exerting a synergistic pro-inflammatory effect in conjunction with infiltrating macrophages. This effect is specifically characterized by the upregulation of M1 polarization markers and the heightened expression of inflammatory factors, such as TNF-α. This process highlights the role of KTRMs in defending against pathogenic bacterial infections and their involvement in transplant rejection. During the recovery phase following injury, KTRMs transform into an anti-inflammatory M2 phenotype, showcasing robust tissue repair capabilities. This indicates that, upon kidney injury, KTRMs can swiftly respond to alterations in the internal environment of the kidney and transform between different phenotypes to perform specific functions. We emphasize the significant immunoregulatory role of KTRMs in the progression of kidney injury and other renal diseases. Many studies have focused on managing kidney injury, inflammation, repair, and fibrosis through the regulation of KTRMs. However, the specific mechanisms by which target molecules regulate KTRMs and the intricate ways in which KTRMs influence various kidney diseases, remain to be elucidated. Furthermore, in the context of kidney diseases, KTRMs are among the first responders to damage signals. In addition to coordinating the recruitment of BMRMs, KTRMs can also enhance inflammatory responses by activating the M1 phenotype, which often results in exacerbated kidney damage. During the renal repair process, KTRMs can synergistically collaborate with BMRMs to promote renal tissue repair. This regulatory interaction between KTRMs and BMRMs offers new insights for the treatment of kidney diseases.

In the renal immune microenvironment, KTRMs play a pivotal role in regulating the infiltrating macrophages and neutrophils, as well as the activation of other immune cells. This underscores the clinical significance of targeting KTRMs to address kidney injury. Furthermore, studies have demonstrated that small molecule drugs aimed at specific KTRM subpopulations can effectively reduce mortality in AKI mouse models and alleviate symptoms of renal fibrosis (55). Such advancements highlight the complex and critical role that KTRMs play in kidney disease. A deeper exploration into the specific mechanisms by which KTRMs contribute to kidney disease, the targeted regulation of their metabolic pathways, and the use of pharmacological agents to modulate KTRM polarization could pave the way for novel therapeutic strategies aimed at improving kidney disease prognosis. Current research on TRMs primarily employs high-throughput cell profiling technologies, such as flow cytometry and single-cell RNA sequencing (104). Additionally, intravital microscopy and magnetic resonance imaging (MRI) are utilized to investigate TRM activity and inflammation, thereby facilitating the study of TRM immune monitoring functions (105, 106). Previous studies have confirmed the existence of a KTRM population co-expressing CD74 and CD81 in both human and rat kidneys (26). Moreover, the development of human induced pluripotent stem cell (iPSC)-derived macrophages has been shown to be associated with TRMs, occurring in a Myb-independent manner and relying on the transcription factors RUNX1 and SPI1 (PU.1) (107). This connection enhances the translational potential of KTRM studies from animal models to human patients and provides new insights into the role of human TRMs in pathology and homeostasis.

## Glossary

TRM: Tissue-resident macrophage
KTRMs: Kidney TRMs
EMPs: erythro-myeloid progenitors
YS: yolk sac
FL: fetal liver
MOs: monocytes
VAT: visceral adipose tissue
TLR: Toll-like receptor
HO-1: heme oxygenase-1
RAGE: receptor for advanced glycation end products
EGFR: epidermal growth factor receptor
BMRMs: bone marrow-derived macrophages
Ahr: Aryl hydrocarbon receptor
Nfatc1 and Nfatc2: nuclear factors of activated T cells 1 and 2
Irf9: interferon regulatory factor 9
TIMD4: T cell immunoglobulin and mucin domain 4
LYVE1: lymphatic endothelial hyaluronic acid receptor 1
FOLR2: folate receptor beta
ICs: immune complexes
scRNAseq: single-cell RNA sequencing
MDP: monocyte-derived precursors
Lysm: lysozyme
FABP5: fatty acid-binding protein 5
MPC: mononuclear phagocyte
MHC II: histocompatibility complex class II
P7: postnatal day 7
E14.5: embryonic day 14.5
TIM-4: T cell immunoglobulin and mucin domain 4
MERTK: MER receptor tyrosine kinase
FcγRs: Fcγ receptors
AKI: acute kidney injury
IRI: ischemia-reperfusion injury
ROS: reactive oxygen species
CLP: cecal ligation and puncture
iRhom2: inactive rhomboid protein 2
FAO: fatty acid oxidation
PG: prostaglandin
ICAM-1: intercellular adhesion molecule-1
UUO: unilateral ureteral obstruction
MFG-E8: milk fat globule epidermal growth factor 8
CI: cold ischemia
HA: heat acclimation
HSP 70: heat shock protein 70
TEC: tubular epithelial cell
CD: collecting duct
TAC: transverse aortic constriction
LCMs: large-cavity macrophages
LN: lupus nephritis
SLE: systemic lupus erythematosus
Tregs: tissue-resident regulatory T cells
iNOS: inducible nitric oxide synthase
CCL: Chemokine (CC-motif) ligand
CCR2: C-C motif chemokine receptor 2
BAFF: B cell activating factor
M−CSF1: Macrophage Colony-Stimulating Factor 1
AGEs: Advanced Glycation End-products
DMAPs: Damage-associated molecular patterns
mtDNA: Mitochondrial DNA
mtROS: mitochondrial reactive oxygen species
MyD88: Myeloid differentiation primary response protein 88
TNF-α: Tumor Necrosis Factor-α
TGF-β1: Transforming Growth Factor Beta 1
Sp1: Specificity Protein 1
Smad: drosophila mothers against decapentaplegic protein
GM-CSF: Granulocyte-macrophage colony-stimulating factor
MCP-1: Monocyte chemoattractant protein-1
IL: Interleukin
IL17ra: Interleukin-17 Receptor A
ccRCC: Clear cell renal cell carcinoma
HAVCR2: Hepatitis A virus cellular receptor 2
LAG3: Lymphocyte-Activation Gene 3
Cxcl: C-X-C motif chemokine ligand
mbCSF1: Macrophage Colony-Stimulating Factor 1
CSF1R: Colony-Stimulating Factor 1 Receptor
SNAT3: Solute Carrier Family 38 Member 3
PEPCK: Phosphoenolpyruvate carboxykinase
OXPHOS: oxidative phosphorylation.

## References

[1] Wynn TA Vannella KM. Macrophages in tissue repair, regeneration, and fibrosis. Immunity. (2016) 44:450–62. doi: 10.1016/j.immuni.2016.02.015 PMC4794754 26982353

[2] Gomez Perdiguero E Klapproth K Schulz C Busch K Azzoni E Crozet L. Tissue-resident macrophages originate from yolk-sac-derived erythro-myeloid progenitors. Nature. (2015) 518:547–51. doi: 10.1038/nature13989 PMC5997177 25470051

[3] Davies LC Jenkins SJ Allen JE Taylor PR. Tissue-resident macrophages. Nat Immunol. (2013) 14:986–95. doi: 10.1038/ni.2705 PMC4045180 24048120

[4] Lazarov T Juarez-Carreno S Cox N Geissmann F. Physiology and diseases of tissue-resident macrophages. Nature. (2023) 618:698–707. doi: 10.1038/s41586-023-06002-x 37344646 PMC10649266

[5] Schulz C Gomez Perdiguero E Chorro L Szabo-Rogers H Cagnard N Kierdorf K. A lineage of myeloid cells independent of myb and hematopoietic stem cells. Science. (2012) 336:86–90. doi: 10.1126/science.1219179 22442384

[6] Varol C Mildner A Jung S. Macrophages: development and tissue specialization. Annu Rev Immunol. (2015) 33:643–75. doi: 10.1146/annurev-immunol-032414-112220 25861979

[7] Hoeffel G Chen J Lavin Y Low D Almeida FF See P. C-myb(+) erythro-myeloid progenitor-derived fetal monocytes give rise to adult tissue-resident macrophages. Immunity. (2015) 42:665–78. doi: 10.1016/j.immuni.2015.03.011 PMC4545768 25902481

[8] Vasamsetti SB Coppin E Zhang X Florentin J Koul S Gotberg M. Apoptosis of hematopoietic progenitor-derived adipose tissue-resident macrophages contributes to insulin resistance after myocardial infarction. Sci Transl Med. (2020) 12:eaaw0638. doi: 10.1126/scitranslmed.aaw0638 32718989 PMC7813555

[9] Devey L Ferenbach D Mohr E Sangster K Bellamy CO Hughes J. Tissue-resident macrophages protect the liver from ischemia reperfusion injury via a heme oxygenase-1-dependent mechanism. Mol Ther. (2009) 17:65–72. doi: 10.1038/mt.2008.237 19002167 PMC2834991

[10] Zhong H Ji J Zhuang J Xiong Z Xie P Liu X. Tissue-resident macrophages exacerbate lung injury after remote sterile damage. Cell Mol Immunol. (2024) 21:332–48. doi: 10.1038/s41423-024-01125-1 PMC10979030 38228746

[11] Bello AK Okpechi IG Levin A Johnson DW. Variations in kidney care management and access: regional assessments of the 2023 international society of nephrology global kidney health atlas (Isn-gkha). Kidney Int Suppl (2011). (2024) 13:1–5. doi: 10.1016/j.kisu.2023.12.001 38619132 PMC11010599

[12] Li G Yang H Zhang D Zhang Y Liu B Wang Y. The role of macrophages in fibrosis of chronic kidney disease. BioMed Pharmacother. (2024) 177:117079. doi: 10.1016/j.biopha.2024.117079 38968801

[13] Zhu Q He J Cao Y Liu X Nie W Han F. Analysis of mononuclear phagocytes disclosed the establishment processes of two macrophage subsets in the adult murine kidney. Front Immunol. (2022) 13:805420. doi: 10.3389/fimmu.2022.805420 35359928 PMC8960422

[14] Mass E Ballesteros I Farlik M Halbritter F Gunther P Crozet L. Specification of tissue-resident macrophages during organogenesis. Science. (2016) 353:aaf4238. doi: 10.1126/science.aaf4238 27492475 PMC5066309

[15] Dick SA Wong A Hamidzada H Nejat S Nechanitzky R Vohra S. Three tissue resident macrophage subsets coexist across organs with conserved origins and life cycles. Sci Immunol. (2022) 7:eabf7777. doi: 10.1126/sciimmunol.abf7777 34995099

[16] Vorsatz C Friedrich N Nimmerjahn F Biburger M. There is strength in numbers: quantitation of fc gamma receptors on murine tissue-resident macrophages. . Int J Mol Sci. (2021) 22:12172. doi: 10.3390/ijms222212172 34830050 PMC8620503

[17] Stamatiades EG Tremblay ME Bohm M Crozet L Bisht K Kao D. Immune monitoring of trans-endothelial transport by kidney-resident macrophages. Cell. (2016) 166:991–1003. doi: 10.1016/j.cell.2016.06.058 27477514 PMC4983224

[18] Yashchenko A Bland SJ Song CJ Ahmed UKB Sharp R Darby IG. Cx3cr1 controls kidney resident macrophage heterogeneity. Front Immunol. (2023) 14:1082078. doi: 10.3389/fimmu.2023.1082078 37256130 PMC10225589

[19] Liu F Dai S Feng D Qin Z Peng X Sakamuri S. Distinct fate, dynamics and niches of renal macrophages of bone marrow or embryonic origins. Nat Commun. (2020) 11:2280. doi: 10.1038/s41467-020-16158-z 32385245 PMC7210253

[20] Lever JM Hull TD Boddu R Pepin ME Black LM Adedoyin OO. Resident Macrophages Reprogram toward a Developmental State after Acute Kidney Injury. JCI Insight. (2019) 4:e125503. doi: 10.1172/jci.insight.125503 30674729 PMC6413788

[21] Lever JM Yang Z Boddu R Adedoyin OO Guo L Joseph R. Parabiosis reveals leukocyte dynamics in the kidney. Lab Invest. (2018) 98:391–402. doi: 10.1038/labinvest.2017.130 29251733 PMC5839939

[22] Zimmerman KA Yang Z Lever JM Li Z Croyle MJ Agarwal A. Kidney resident macrophages in the rat have minimal turnover and replacement by blood monocytes. Am J Physiol Renal Physiol. (2021) 321:F162–F9. doi: 10.1152/ajprenal.00129.2021 PMC8424665 34180717

[23] Lei T Zhang J Zhang Q Ma X Xu Y Zhao Y. Defining newly formed and tissue-resident bone marrow-derived macrophages in adult mice based on lysozyme expression. Cell Mol Immunol. (2022) 19:1333–46. doi: 10.1038/s41423-022-00936-4 PMC9708686 36348079

[24] Jenkins SJ Ruckerl D Cook PC Jones LH Finkelman FD van Rooijen N. Local macrophage proliferation, rather than recruitment from the blood, is a signature of th2 inflammation. Science. (2011) 332:1284–8. doi: 10.1126/science.1204351 PMC3128495 21566158

[25] Kawakami T Lichtnekert J Thompson LJ Karna P Bouabe H Hohl TM. Resident renal mononuclear phagocytes comprise five discrete populations with distinct phenotypes and functions. J Immunol. (2013) 191:3358–72. doi: 10.4049/jimmunol.1300342 PMC3808972 23956422

[26] Zimmerman KA Bentley MR Lever JM Li Z Crossman DK Song CJ. Single-cell rna sequencing identifies candidate renal resident macrophage gene expression signatures across species. J Am Soc Nephrol. (2019) 30:767–81. doi: 10.1681/asn.2018090931 PMC6493978 30948627

[27] Salei N Rambichler S Salvermoser J Papaioannou NE Schuchert R Pakalniskyte D. The kidney contains ontogenetically distinct dendritic cell and macrophage subtypes throughout development that differ in their inflammatory properties. J Am Soc Nephrol. (2020) 31:257–78. doi: 10.1681/ASN.2019040419 PMC7003301 31932472

[28] Chew C Brand OJ Yamamura T Lawless C Morais M Zeef L. Kidney resident macrophages have distinct subsets and multifunctional roles. Matrix Biol. (2024) 127:23–37. doi: 10.1016/j.matbio.2024.02.002 38331051 PMC7619362

[29] McEvoy CM Murphy JM Zhang L Clotet-Freixas S Mathews JA An J. Single-cell profiling of healthy human kidney reveals features of sex-based transcriptional programs and tissue-specific immunity. Nat Commun. (2022) 13:7634. doi: 10.1038/s41467-022-35297-z 36496458 PMC9741629

[30] Munro DAD Wineberg Y Tarnick J Vink CS Li Z Pridans C. Macrophages restrict the nephrogenic field and promote endothelial connections during kidney development. Elife. (2019) 8:e43271. doi: 10.7554/eLife.43271 30758286 PMC6374076

[31] Dai XM Ryan GR Hapel AJ Dominguez MG Russell RG Kapp S. Targeted disruption of the mouse colony-stimulating factor 1 receptor gene results in osteopetrosis, mononuclear phagocyte deficiency, increased primitive progenitor cell frequencies, and reproductive defects. Blood. (2002) 99:111–20. doi: 10.1182/blood.v99.1.111 11756160

[32] Rae F Woods K Sasmono T Campanale N Taylor D Ovchinnikov DA. Characterisation and trophic functions of murine embryonic macrophages based upon the use of a csf1r-egfp transgene reporter. Dev Biol. (2007) 308:232–46. doi: 10.1016/j.ydbio.2007.05.027 17597598

[33] Bellomo R Kellum JA Ronco C. Acute kidney injury. Lancet. (2012) 380:756–66. doi: 10.1016/S0140-6736(11)61454-2 22617274

[34] Schetz M Prowle J. Focus on acute kidney injury 2017. Intensive Care Med. (2018) 44:1992–4. doi: 10.1007/s00134-018-5357-8 30187113

[35] Sabapathy V Price A Cheru NT Venkatadri R Dogan M Costlow G. St2+ T-regulatory cells in renal inflammation and fibrosis following ischemic kidney injury. J Am Soc Nephrol. (2024) 36:73–86. doi: 10.1681/ASN.0000000000000471 PMC11706559 39186386

[36] Wang X Xie X Ni JY Li JY Sun XA Xie HY. Usp11 promotes renal tubular cell pyroptosis and fibrosis in uuo mice via inhibiting klf4 ubiquitin degradation. Acta Pharmacol Sin. (2024) 46:159–70. doi: 10.1038/s41401-024-01363-z PMC11696738 39147900

[37] Blanco-Gozalo V Quiros Y Vicente-Vicente L Casanova AG Sancho-Martinez SM Lopez-Hernandez FJ. Urinary gm2ap coincides with renal cortical damage and grades cisplatin nephrotoxicity severity in rats. Toxicology. (2024) 508:153919. doi: 10.1016/j.tox.2024.153919 39137829

[38] Chang YM Chou YT Kan WC Shiao CC. Sepsis and acute kidney injury: A review focusing on the bidirectional interplay. Int J Mol Sci. (2022) 23:9159. doi: 10.3390/ijms23169159 36012420 PMC9408949

[39] Privratsky JR Ide S Chen Y Kitai H Ren J Fradin H. A macrophage-endothelial immunoregulatory axis ameliorates septic acute kidney injury. Kidney Int. (2023) 103:514–28. doi: 10.1016/j.kint.2022.10.008 PMC9974788 36334787

[40] Baatarjav C Komada T Karasawa T Yamada N Sampilvanjil A Matsumura T. Dsdna-induced aim2 pyroptosis halts aberrant inflammation during rhabdomyolysis-induced acute kidney injury. Cell Death Differ. (2022) 29:2487–502. doi: 10.1038/s41418-022-01033-9 PMC9750976 35739254

[41] Cheung MD Erman EN Moore KH Lever JM Li Z LaFontaine JR. Resident macrophage subpopulations occupy distinct microenvironments in the kidney. JCI Insight. (2022) 7:e161078. doi: 10.1172/jci.insight.161078 36066976 PMC9714795

[42] Zhang YL Tang TT Wang B Wen Y Feng Y Yin Q. Identification of a novel ecm remodeling macrophage subset in aki to ckd transition by integrative spatial and single-cell analysis. Adv Sci (Weinh). (2024) 11:e2309752. doi: 10.1002/advs.202309752 39119903 PMC11481374

[43] Xu S Shinohara ML. Tissue-resident macrophages in fungal infections. Front Immunol. (2017) 8:1798. doi: 10.3389/fimmu.2017.01798 29312319 PMC5732976

[44] Teo YJ Ng SL Mak KW Setiagani YA Chen Q Nair SK. Renal cd169(++) resident macrophages are crucial for protection against acute systemic candidiasis. . Life Sci Alliance. (2021) 4:e202000890. doi: 10.26508/lsa.202000890 33608410 PMC7918719

[45] Jawhara S Pluskota E Cao W Plow EF Soloviev DA Deepe GS. Distinct effects of integrins αxβ2and αmβ2on leukocyte subpopulations during inflammation and antimicrobial responses. Infection Immun. (2017) 85:e00644-16. doi: 10.1128/iai.00644-16 PMC5203657 27799334

[46] Sun J Ge X Wang Y Niu L Tang L Pan S. Usf2 knockdown downregulates thbs1 to inhibit the tgf-beta signaling pathway and reduce pyroptosis in sepsis-induced acute kidney injury. Pharmacol Res. (2022) 176:105962. doi: 10.1016/j.phrs.2021.105962 34756923

[47] Ito S Goto H Tanoue K Koiwai K Ishikiriyama T Kearney BM. Early treatment with C-reactive protein-derived peptide reduces septic acute kidney injury in mice via controlled activation of kidney macrophages. J Leukoc Biol. (2023) 113:400–13. doi: 10.1093/jleuko/qiad015 36802006

[48] Xu MX Qin YT Ge CX Gu TT Lou DS Li Q. Activated irhom2 drives prolonged pm(2.5) exposure-triggered renal injury in nrf2-defective mice. Nanotoxicology. (2018) 12:1045–67. doi: 10.1080/17435390.2018.1513093 30257117

[49] Goto H Nakashima H Mori K Tanoue K Ito S Kearney BM. L-carnitine pretreatment ameliorates heat stress-induced acute kidney injury by restoring mitochondrial function of tubular cells. Am J Physiol Renal Physiol. (2024) 326:F338–f51. doi: 10.1152/ajprenal.00196.2023 38095023

[50] Zhang MZ Yao B Yang S Jiang L Wang S Fan X. Csf-1 signaling mediates recovery from acute kidney injury. J Clin Invest. (2012) 122:4519–32. doi: 10.1172/JCI60363 PMC3533529 23143303

[51] Lee S Huen S Nishio H Nishio S Lee HK Choi BS. Distinct macrophage phenotypes contribute to kidney injury and repair. J Am Soc Nephrol. (2011) 22:317–26. doi: 10.1681/ASN.2009060615 PMC3029904 21289217

[52] Wang Y Chang J Yao B Niu A Kelly E Breeggemann MC. Proximal tubule-derived colony stimulating factor-1 mediates polarization of renal macrophages and dendritic cells, and recovery in acute kidney injury. Kidney Int. (2015) 88:1274–82. doi: 10.1038/ki.2015.295 PMC4675680 26422503

[53] Karasawa K Asano K Moriyama S Ushiki M Monya M Iida M. Vascular-resident cd169-positive monocytes and macrophages control neutrophil accumulation in the kidney with ischemia-reperfusion injury. J Am Soc Nephrol. (2015) 26:896–906. doi: 10.1681/ASN.2014020195 25266072 PMC4378108

[54] Ji X Cai J Liang L Shi T Liu J. Gene expression variability across cells and species shapes the relationship between renal resident macrophages and infiltrated macrophages. BMC Bioinf. (2023) 24:72. doi: 10.1186/s12859-023-05198-z PMC9976410 36858955

[55] Yao W Chen Y Li Z Ji J You A Jin S. Single cell rna sequencing identifies a unique inflammatory macrophage subset as a druggable target for alleviating acute kidney injury. Adv Sci (Weinh). (2022) 9:e2103675. doi: 10.1002/advs.202103675 35112806 PMC9036000

[56] Park JG Lee CR Kim MG Kim G Shin HM Jeon YH. Kidney residency of vista-positive macrophages accelerates repair from ischemic injury. Kidney Int. (2020) 97:980–94. doi: 10.1016/j.kint.2019.11.025 32143848

[57] Brissette MJ Laplante P Qi S Latour M Cailhier JF. Milk fat globule epidermal growth factor-8 limits tissue damage through inflammasome modulation during renal injury. J Leukoc Biol. (2016) 100:1135–46. doi: 10.1189/jlb.3A0515-213RR 27260955

[58] Einecke G Mengel M Hidalgo L Allanach K Famulski KS Halloran PF. The early course of kidney allograft rejection: defining the time when rejection begins. Am J Transplant. (2009) 9:483–93. doi: 10.1111/j.1600-6143.2008.02546.x 19260832

[59] Qi F Adair A Ferenbach D Vass DG Mylonas KJ Kipari T. Depletion of cells of monocyte lineage prevents loss of renal microvasculature in murine kidney transplantation. Transplantation. (2008) 86:1267–74. doi: 10.1097/TP.0b013e318188d433 19005409

[60] Aiello S Podesta MA Rodriguez-Ordonez PY Pezzuto F Azzollini N Solini S. Transplantation-induced ischemia-reperfusion injury modulates antigen presentation by donor renal cd11c(+)F4/80(+) macrophages through il-1r8 regulation. J Am Soc Nephrol. (2020) 31:517–31. doi: 10.1681/ASN.2019080778 PMC7062225 31988271

[61] Goto H Nakashima M Nakashima H Noguchi M Imakiire T Oshima N. Heat acclimation ameliorated heat stress-induced acute kidney injury and prevented changes in kidney macrophages and fibrosis. Am J Physiol Renal Physiol. (2022) 323:F243–F54. doi: 10.1152/ajprenal.00065.2022 PMC9394728 35796461

[62] Stewart BJ Ferdinand JR Young MD Mitchell TJ Loudon KW Riding AM. Spatiotemporal immune zonation of the human kidney. Science. (2019) 365:1461–6. doi: 10.1126/science.aat5031 PMC7343525 31604275

[63] Saavedra PHV Perry JSA. Kidney macrophages tap the stream. Immunity. (2024) 57:3–5. doi: 10.1016/j.immuni.2023.12.008 38198853

[64] He J Cao Y Zhu Q Wang X Cheng G Wang Q. Renal macrophages monitor and remove particles from urine to prevent tubule obstruction. Immunity. (2024) 57:106–23.e7. doi: 10.1016/j.immuni.2023.12.003 38159573

[65] Fujiu K Shibata M Nakayama Y Ogata F Matsumoto S Noshita K. A heart-brain-kidney network controls adaptation to cardiac stress through tissue macrophage activation. Nat Med. (2017) 23:611–22. doi: 10.1038/nm.4326 28394333

[66] Finlay CM Parkinson JE Zhang L Chan BHK Ajendra J Chenery A. T helper 2 cells control monocyte to tissue-resident macrophage differentiation during nematode infection of the pleural cavity. Immunity. (2023) 56:1064–81.e10. doi: 10.1016/j.immuni.2023.02.016 36948193 PMC7616141

[67] Duffield JS Tipping PG Kipari T Cailhier JF Clay S Lang R. Conditional ablation of macrophages halts progression of crescentic glomerulonephritis. Am J Pathol. (2005) 167:1207–19. doi: 10.1016/S0002-9440(10)61209-6 PMC1603796 16251406

[68] Liang CL Wei YY Chen Y Luo Y Qin F Chen Y. Zhen-wu-tang ameliorates lupus nephritis by diminishing renal tissue-resident memory cd8(+) T cells via suppressing il-15/stat3 pathway. BioMed Pharmacother. (2024) 174:116597. doi: 10.1016/j.biopha.2024.116597 38643544

[69] Suchanek O Ferdinand JR Tuong ZK Wijeyesinghe S Chandra A Clauder AK. Tissue-resident B cells orchestrate macrophage polarisation and function. Nat Commun. (2023) 14:7081. doi: 10.1038/s41467-023-42625-4 37925420 PMC10625551

[70] Lindquist JA Hildebrandt J Philipsen L Mertens PR. Immune complexes and complexity: investigating mechanisms of renal disease. Int Urol Nephrol. (2017) 49:735–9. doi: 10.1007/s11255-016-1450-5 27864659

[71] Richoz N Tuong ZK Loudon KW Patino-Martinez E Ferdinand JR Portet A. Distinct pathogenic roles for resident and monocyte-derived macrophages in lupus nephritis. JCI Insight. (2022) 7:e159751. doi: 10.1172/jci.insight.159751 36345939 PMC9675473

[72] Zhou C Bai X Yang Y Shi M Bai XY. Single-cell sequencing informs that mesenchymal stem cell alleviates renal injury through regulating kidney regional immunity in lupus nephritis. Stem Cells Dev. (2023) 32:465–83. doi: 10.1089/scd.2023.0047 37082951

[73] Aizawa M Suzuki Y Suzuki H Pang H Kihara M Nakata J. Uncoupling of glomerular iga deposition and disease progression in alymphoplasia mice with iga nephropathy. PloS One. (2014) 9:e95365. doi: 10.1371/journal.pone.0095365 24743510 PMC3990643

[74] Watanabe M Kaneko Y Ohishi Y Kinoshita M Sakairi T Ikeuchi H. Importance of methodology in the evaluation of renal mononuclear phagocytes and analysis of a model of experimental nephritis with shp1 conditional knockout mice. Biochem Biophys Rep. (2020) 22:100741. doi: 10.1016/j.bbrep.2020.100741 32154390 PMC7057148

[75] Ito S Nakashima H Ishikiriyama T Nakashima M Yamagata A Imakiire T. Effects of a ccr2 antagonist on macrophages and toll-like receptor 9 expression in a mouse model of diabetic nephropathy. Am J Physiol Renal Physiol. (2021) 321:F757–F70. doi: 10.1152/ajprenal.00191.2021 34719947

[76] Jianbin X Peng D Jing Z Xiaofei A Yudie F Jing Z. (5r)-5-hydroxytriptolide ameliorates diabetic kidney damage by inhibiting macrophage infiltration and its cross-talk with renal resident cells. Int Immunopharmacol. (2024) 126:111253. doi: 10.1016/j.intimp.2023.111253 38007850

[77] Ito S Nakashima M Ishikiriyama T Nakashima H Yamagata A Imakiire T. Effects of L-carnitine treatment on kidney mitochondria and macrophages in mice with diabetic nephropathy. Kidney Blood Press Res. (2022) 47:277–90. doi: 10.1159/000522013 35104825

[78] Fu J Sun Z Wang X Zhang T Yuan W Salem F. The single-cell landscape of kidney immune cells reveals transcriptional heterogeneity in early diabetic kidney disease. Kidney Int. (2022) 102:1291–304. doi: 10.1016/j.kint.2022.08.026 PMC9691617 36108806

[79] Kim KH Park JH Lee WR Park JS Kim HC Park KK. The inhibitory effect of chimeric decoy oligodeoxynucleotide against nf-kappab and sp1 in renal interstitial fibrosis. J Mol Med (Berl). (2013) 91:573–86. doi: 10.1007/s00109-012-0972-2 23114611

[80] Lin SL Castano AP Nowlin BT Lupher ML Jr. Duffield JS. Bone marrow ly6chigh monocytes are selectively recruited to injured kidney and differentiate into functionally distinct populations. J Immunol. (2009) 183:6733–43. doi: 10.4049/jimmunol.0901473 19864592

[81] Zhang G Thomas AL Marshall AL Kernan KA Su Y Zheng Y. Nicotinic acetylcholine receptor alpha1 Promotes Calpain-1 Activation and Macrophage Inflammation in Hypercholesterolemic Nephropathy. Lab Invest. (2011) 91:106–23. doi: 10.1038/labinvest.2010.135 PMC3188436 20661225

[82] Sears SM Vega AA Kurlawala Z Oropilla GB Krueger A Shah PP. F4/80(Hi) Resident Macrophages Contribute to Cisplatin-Induced Renal Fibrosis. Kidney360. (2022) 3:818–33. doi: 10.34067/KID.0006442021 PMC9438415 36128491

[83] Xu L Sharkey D Cantley LG. Tubular Gm-Csf Promotes Late Mcp-1/Ccr2-Mediated Fibrosis and Inflammation after Ischemia/Reperfusion Injury. J Am Soc Nephrol. (2019) 30:1825–40. doi: 10.1681/ASN.2019010068 PMC6779361 31315923

[84] Ge S Hertel B Susnik N Rong S Dittrich AM Schmitt R. Interleukin 17 Receptor a Modulates Monocyte Subsets and Macrophage Generation *in Vivo* . PloS One. (2014) 9:e85461. doi: 10.1371/journal.pone.0085461 24454873 PMC3893198

[85] Shi Y Zhang Q Bi H Lu M Tan Y Zou D. Decoding the Multicellular Ecosystem of Vena Caval Tumor Thrombus in Clear Cell Renal Cell Carcinoma by Single-Cell Rna Sequencing. Genome Biol. (2022) 23:87. doi: 10.1186/s13059-022-02651-9 35361264 PMC8969307

[86] Krishna C DiNatale RG Kuo F Srivastava RM Vuong L Chowell D. Single-Cell Sequencing Links Multiregional Immune Landscapes and Tissue-Resident T Cells in Ccrcc to Tumor Topology and Therapy Efficacy. Cancer Cell. (2021) 39:662–77 e6. doi: 10.1016/j.ccell.2021.03.007 33861994 PMC8268947

[87] Brech D Herbstritt AS Diederich S Straub T Kokolakis E Irmler M. Dendritic Cells or Macrophages? The Microenvironment of Human Clear Cell Renal Cell Carcinoma Imprints a Mosaic Myeloid Subtype Associated with Patient survival. Cells. (2022) 11:3289. doi: 10.3390/cells11203289 36291154 PMC9600747

[88] Xu W Ye S Liu W Guo H Zhang L Wei S. Single-cell rna-seq analysis decodes the kidney microenvironment induced by polystyrene microplastics in mice receiving a high-fat diet. J Nanobiotechnology. (2024) 22:13. doi: 10.1186/s12951-023-02266-7 38167034 PMC10762848

[89] Raghubar AM Matigian NA Crawford J Francis L Ellis R Healy HG. High risk clear cell renal cell carcinoma microenvironments contain protumour immunophenotypes lacking specific immune checkpoints. NPJ Precis Oncol. (2023) 7:88. doi: 10.1038/s41698-023-00441-5 37696903 PMC10495390

[90] Fakhouri F Zuber J Fremeaux-Bacchi V Loirat C. Haemolytic uraemic syndrome. Lancet. (2017) 390:681–96. doi: 10.1016/S0140-6736(17)30062-4 28242109

[91] Lill JK Thiebes S Pohl JM Bottek J Subramaniam N Christ R. Tissue-resident macrophages mediate neutrophil recruitment and kidney injury in shiga toxin-induced hemolytic uremic syndrome. Kidney Int. (2021) 100:349–63. doi: 10.1016/j.kint.2021.03.039 33930412

[92] Zimmerman KA Song CJ Li Z Lever JM Crossman DK Rains A. Tissue-resident macrophages promote renal cystic disease. J Am Soc Nephrol. (2019) 30:1841–56. doi: 10.1681/ASN.2018080810 PMC6779366 31337691

[93] Sedaka R Huang J Yamaguchi S Lovelady C Hsu JS Shinde S. Accelerated Cystogenesis by Dietary Protein Load Is Dependent on, but Not Initiated by Kidney Macrophages. Front Med (Lausanne). (2023) 10:1173674. doi: 10.3389/fmed.2023.1173674 37538309 PMC10394241

[94] Sung CYW Hayase N Yuen PST Lee J Fernandez K Hu X. Macrophage depletion protects against cisplatin-induced ototoxicity and nephrotoxicity. Sci Adv. (2024) 10:eadk9878. doi: 10.1126/sciadv.adk9878 39047106 PMC11268410

[95] Rojo R Raper A Ozdemir DD Lefevre L Grabert K Wollscheid-Lengeling E. Deletion of a csf1r enhancer selectively impacts csf1r expression and development of tissue macrophage populations. Nat Commun. (2019) 10:3215. doi: 10.1038/s41467-019-11053-8 31324781 PMC6642117

[96] Liu Z Lv R Guo H Zhang B Wang X Qiang P. Proliferation of renal macrophage via mr/csf1 pathway induced with aldosterone and inhibited by esaxerenone. Int Immunopharmacol. (2025) 149:114208. doi: 10.1016/j.intimp.2025.114208 39923576

[97] Dangi A Husain I Jordan CZ Yu S Natesh N Shen X. Blocking ccl8-ccr8-mediated early allograft inflammation improves kidney transplant function. J Am Soc Nephrol. (2022) 33:1876–90. doi: 10.1681/asn.2022020139 PMC9528333 35973731

[98] Ma Q Xu M Jing X Qiu J Huang S Yan H. Honokiol suppresses the aberrant interactions between renal resident macrophages and tubular epithelial cells in lupus nephritis through the nlrp3/il-33/st2 axis. Cell Death Dis. (2023) 14:174. doi: 10.1038/s41419-023-05680-9 36859530 PMC9977833

[99] do Valle Duraes F Lafont A Beibel M Martin K Darribat K Cuttat R. Immune cell landscaping reveals a protective role for regulatory T cells during kidney injury and fibrosis. JCI Insight. (2020) 5:e130651. doi: 10.1172/jci.insight.130651 32051345 PMC7098794

[100] Zylberberg AK Cottle DL Runting J Rodrigues G Tham MS Jones LK. Modulating inflammation with interleukin 37 treatment ameliorates murine autosomal dominant polycystic kidney disease. Kidney Int. (2024) 105:731–43. doi: 10.1016/j.kint.2023.12.006 38158181

[101] Luan J Fu J Chen C Jiao C Kong W Zhang Y. Lna-anti-mir-150 ameliorated kidney injury of lupus nephritis by inhibiting renal fibrosis and macrophage infiltration. Arthritis Res Ther. (2019) 21:276. doi: 10.1186/s13075-019-2044-2 31829247 PMC6907329

[102] Zheng Y Lu P Deng Y Wen L Wang Y Ma X. Single-cell transcriptomics reveal immune mechanisms of the onset and progression of iga nephropathy. Cell Rep. (2020) 33:108525. doi: 10.1016/j.celrep.2020.108525 33357427

[103] Emal D Rampanelli E Stroo I Butter LM Teske GJ Claessen N. Depletion of gut microbiota protects against renal ischemia-reperfusion injury. J Am Soc Nephrol. (2017) 28:1450–61. doi: 10.1681/asn.2016030255 PMC5407717 27927779

[104] Alonso-Herranz L Porcuna J Ricote M. Isolation and purification of tissue resident macrophages for the analysis of nuclear receptor activity. Methods Mol Biol. (2019) 1951:59–73. doi: 10.1007/978-1-4939-9130-3_5 30825144

[105] Hublitz KW Stamatiades EG. Elucidating immune monitoring of tissue-resident macrophages by intravital microscopy. Methods Mol Biol. (2024) 2713:337–46. doi: 10.1007/978-1-0716-3437-0_23 37639134

[106] Stirrat CG Alam SR MacGillivray TJ Gray CD Forsythe R Dweck MR. Ferumoxytol-enhanced magnetic resonance imaging methodology and normal values at 1.5 and 3t. J Cardiovasc Magn Reson. (2016) 18:46. doi: 10.1186/s12968-016-0261-2 27465647 PMC4964058

[107] Buchrieser J James W Moore MD. Human induced pluripotent stem cell-derived macrophages share ontogeny with myb-independent tissue-resident macrophages. Stem Cell Rep. (2017) 8:334–45. doi: 10.1016/j.stemcr.2016.12.020 PMC5312255 28111278

